# Response to Authors' response

**DOI:** 10.1111/1556-4029.70352

**Published:** 2026-05-07

**Authors:** Aaron Olson

**Affiliations:** ^1^ ARO Consulting LLC Hugo USA

1

See Original Article *here*

See Commentary on *here*

See Authors' response *here*

Editor,

I appreciate the authors' reply to my Commentary and their willingness to clarify important points. However, several scientific issues remain unresolved and merit further clarification for readers.

## 1 GENERALIZATION BEYOND THE EVALUATED SIMULATOR

The authors respond that they have “no reason to believe” that other conforming commercial simulators would perform differently, provided temperature stability is achieved. This conclusion is not supported by the data presented. Without testing this assertion, one cannot conclude that all simulators will deplete ethanol in the same manner.

## 2 HEADSPACE PARTITION RATIO CLARIFICATION

The authors' reply conflates blood‐to‐breath conversion ratios with air‐to‐water partitioning of ethanol. These are distinct physiological and physicochemical differences and should not be treated as interchangeable.

The authors state that “the solution used in this study indeed contained a ratio of 1.21 g/L of ethanol in water.” That is not a ratio, but a liquid‐phase concentration expressed in specific units.

The vapor concentration above the simulator solution is not determined by this concentration alone, nor by the blood‐to‐breath conversion ratio of 2100:1. Rather, the vapor‐phase ethanol concentration is determined by the assumed water–air (*K*_w/a_) partition coefficient for ethanol, as described by Dubowski, Gullberg, Harger, Jones, and others [1, 2, 3, 4, 5].

The assumed water–air partition coefficient used by the investigators is important because it determines the initial ethanol vapor concentration above the simulator solution at 34°C.

To determine the assumed vapor concentration above a simulator solution headspace, one can use the following equation:

(1) $$C_{\text{vapor}\;\left(\frac{g}{210\;L}\right)}=\frac{C_{\text{solution}\;\left(\frac{g}{L}\right)}}{K_{w/a}}*210$$

where $C_{\text{solution}}$ = ethanol concentration in the simulator solution (g/L); $K_{w/a}\left(34^{\circ }C\right)$ = water‐to‐air partition coefficient for ethanol at 34°C; $C_{\text{vapor}}$ = assumed ethanol vapor concentration in grams per 210 L of air.

**Example:**

$$C_{\text{vapor}\;\left(\frac{g}{210L}\right)}=\frac{1.21\;\left(\frac{g}{L}\right)}{2539}*210=0.100$$

Table 1 illustrates the concept using a simulator with an initial ethanol in water concentration of 1.21 g/L.

**TABLE 1:** Assumed ethanol vapor concentrations derived from a 1.21 g/L simulator solution using various water–air partition coefficients at 34°C.

Simulator solution concentration (g/L)	*K*_w/a_ at 34°C	Assumed vapor concentration (g/210 L)	References
1.21	2539	0.100	Harger et al. [4]
1.21	2572	0.098	Dubowski [1]
1.21	2586	0.098	Jones [2]
1.21	2593	0.097	Gullberg [3]

The authors clarify that they used Harger's air–water partition coefficient of 2539 and cite Gullberg's conclusion that the data were reliable when properly analyzed. However, Gullberg's central finding was that a reanalysis of Harger's data, using appropriate nonlinear regression, yields partition ratios closer to those reported by Dubowski and Jones, and that using Harger's historical value introduces an approximately 2.5% bias.

The authors characterize a 2.5% bias associated with headspace partition ratio selection as acceptable. However, in contexts such as NHTSA calibration unit requirements, where allowable bias is limited to 2% or 0.002 g/210 L, a 2.5% systematic bias is significant and must be explicitly acknowledged and accounted for [6].

The authors state that “Different legislations might prefer to use different partition ratios, but in this case, again, we used Harger's original ratio.” The headspace ratio selected is a scientific question, not a legislative one. From a forensic standpoint, the more conservative numbers reported by Dubowski, Jones, and Gullberg should be used as a starting point to avoid biasing results and overreporting breath alcohol concentrations.

## 3 DATA INTERPRETATION

The authors' reliance on correlation coefficients to justify changes to analytical procedures during the study is inappropriate. The decision to introduce a new quantification method during the ethanol depletion verification introduced a confounding variable that may have biased the data.

## 4 CONCLUSION

I thank the authors for their important work and thoughtful reply. I hope this response helps clarify the issues at hand.

## CONFLICT OF INTEREST STATEMENT

The author is a forensic toxicology consultant who provides case review, expert consultation, and expert testimony in matters involving alcohol and drug testing, including breath alcohol analysis.

## Data Availability

Data sharing is not applicable to this article as no datasets were generated or analyzed during the current study.
